# Capping Agents Enable Well-Dispersed and Colloidally Stable Metallic Magnesium Nanoparticles

**DOI:** 10.1021/acs.jpcc.4c00366

**Published:** 2024-03-12

**Authors:** Thomas M. R. Wayman, Vladimir Lomonosov, Emilie Ringe

**Affiliations:** † Department of Materials Science and Metallurgy, University of Cambridge, 27 Charles Babbage Road, Cambridge CB3 0FS, United Kingdom; ‡ Department of Earth Sciences, University of Cambridge, Downing Street, Cambridge CB2 3EQ, United Kingdom

## Abstract

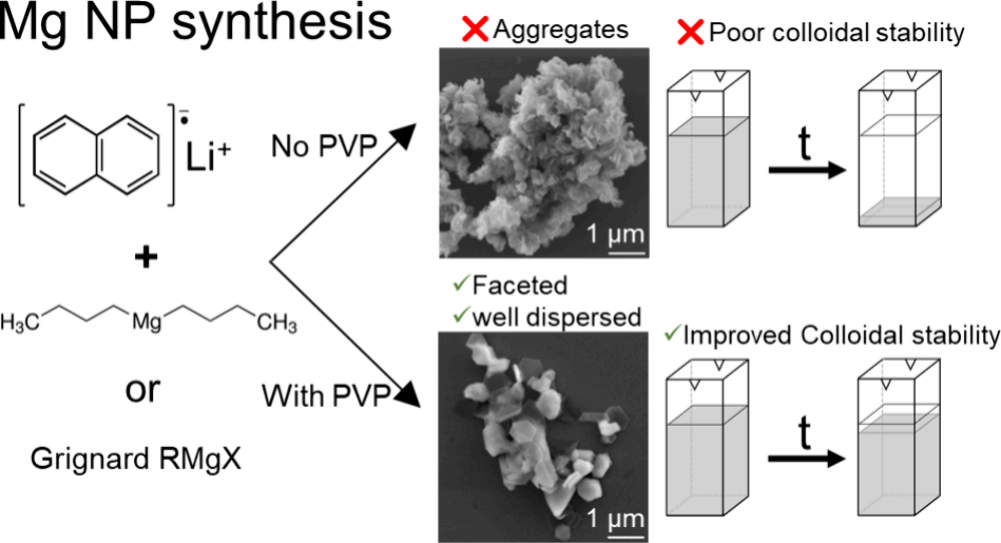

Mg nanoparticles are an emerging plasmonic material due to Mg’s abundance and ability to sustain size- and shape-dependent localized surface plasmon resonances across a broad range of wavelengths from the ultraviolet to the near infrared. However, Mg nanoparticles are colloidally unstable due to their tendency to aggregate and sediment. Nanoparticle aggregation can be inhibited by the addition of capping agents that impart surface charges or steric repulsion. Here, we report that the common capping agents poly(vinyl) pyrrolidone (PVP), polyethylene glycol (PEG), cetyltrimethylammonium bromide (CTAB), and sodium dodecyl sulfate (SDS) interact differently and have varied effects on the aggregation and colloidal stability of Mg nanoparticles. Nanoparticles synthesized in the presence of PVP showed improvements in colloidal stability and reduced aggregation, as observed by electron microscopy and optical spectroscopy. The binding of PVP was confirmed through infrared and X-ray photoelectron spectroscopy. The influence of PVP on the reduction of di-*n*-butyl magnesium was evaluated through analysis of particle size distribution and Mg yield as a function of reaction time, reducing agent, and temperature. Furthermore, the presence of PVP drastically changes the growth pattern of metallic Mg structures obtained from the reduction of the Grignard reagents butylmagnesium chloride and phenylmagnesium chloride by lithium naphthalenide: large polycrystalline aggregates and well-separated faceted nanoparticles grow without and with PVP, respectively. This study provides new synthetic routes that generate colloidally stable and well-dispersed Mg nanoparticles for plasmonic and other applications.

## Introduction

Nanoparticles (NPs) of some metals can sustain localized surface plasmon resonances (LSPRs), a coherent oscillation of conduction electrons leading to energy absorption and strong electric field enhancement.^1^ This absorption, and subsequent decay products including energetic charge carriers and heat, has recently been utilized for photothermal cancer therapy^2^ and to drive a variety of chemical reactions at the NP’s surface.^3−6^ Furthermore, the field enhancement is at the basis of enhanced spectroscopies such as surface-enhanced Raman scattering^7,8^ (SERS) and enables a variety of chemical^9,10^ and biological^11,12^ sensing platforms.

LSPRs are size-, shape-, and composition-dependent. Historically, research into plasmonic NPs has been focused on the noble metals Au and Ag; however, more earth-abundant, cheaper, and differently tunable materials have emerged as alternatives.^13−23^ An example is Mg—an attractive plasmonic metal—due to its ability to sustain resonances across the ultraviolet–visible-near-infrared (UV–vis-NIR) range as well as its position as the eighth most abundant element in the Earth’s crust.^24^

Work on colloidal syntheses of Mg dates back to Rieke and Bales,^25^ who produced Mg powders via the reduction of MgCl_2_ by K in tetrahydrofuran (THF) to form reactive “activated magnesium”. Rieke et al. then further modified the synthesis by introducing a catalytic amount of the electron carrier naphthalene, which enabled reduction of MgCl_2_ by Li et al. at room temperature.^26^ Organomagnesium precursors have also been shown to produce metallic structures, mainly in the form of large networks of aggregates.^27,28^ Reduction of magnesocene (MgCp_2_) has also been reported in dimethoxyethane.^29^

The syntheses of Mg NPs via colloidal reduction of di-*n*-butyl magnesium (MgBu_2_) with lithium naphthalenide, phenanthride, and biphenylide have previously been demonstrated.^28,30−33^ Such syntheses produce a variety of NP shapes, notably single-crystal hexagonal platelets, elongated rod-like structures, and singly twinned structures.^34^ Controlling the mean size of the resulting NPs was made possible by varying reaction parameters such as temperature, concentration, electron carrier, and presence of metal salt additives.^30^ Further control coupled with a narrow (<10% size polydispersity) size distribution was obtained using a seed-mediated approach.^31^

We have demonstrated that Mg NPs have a metallic core using X-ray diffraction (XRD),^30,33^ electron diffraction (ED),^33,34^ and electron energy loss spectroscopy (EELS) of the bulk metal plasmon^30,31,34−37^ and Mg K-edge.^33^ An oxide shell of ∼10 nm in thickness spontaneously forms, as measured by scanning transmission electron microscopy EELS (STEM-EELS),^31,33−35^ STEM-energy-dispersive X-ray spectroscopy (STEM-EDS),^31,34−36^ and atomic resolution TEM.^37^ The shell prevents further oxidation in air and organic solvents, as shown by SEM and XRD.^33^

Despite rapid advances in the synthesis and understanding of Mg NPs, their colloidal stability remains a challenge: Mg NPs aggregate during and after synthesis, leading to rapid sedimentation that alters the suspension’s optical properties. In most plasmonic NP systems, this behavior can be prevented by the addition of a steric or electrostatic barrier, usually in the form of a molecular layer. For Au NPs, for instance, the use of thiols (e.g., dodecanethiol^38,39^), ammonium ions (e.g., cetyltrimethylammonium bromide^40,41^ (CTAB)), and conjugate bases of molecular acids (e.g., citrate^41−43^) has been documented extensively. Furthermore, CTAB is commonly employed as a shape-directing agent in Au nanorod syntheses^44,45^ and can impart colloidal stability due to the formation of a bilayer at the NP-liquid surface.^46^ Meanwhile, sodium dodecyl sulfate (SDS), polyethylene glycol (PEG), and poly(vinyl) pyrrolidone (PVP) have been shown to suppress aggregation of Ag NPs^47^ while PVP has been used as a shape-directing agent leading to the formation of Ag,^48^ Pt,^49^ and Cu^50^ cubes.

Mg NP syntheses involving the presence of molecules other than the Mg precursor and reducing agent have also been reported. Mg nanofibers were synthesized by reduction of MgBu_2_ by Ca in THF in the presence of dodecanethiol,^51^ while Mg nanoflowers have been reported from reduction of MgBu_2_ with lithium naphthalenide in the presence of hexadecylamine.^32^ Furthermore, tetrabutylammonium bromide has been used during electrolysis of a Mg ribbon to a fine Mg-containing powder.^52^ In all of these cases, neither the binding of the added molecules on the surface of the NP nor their effects on colloidal stability have been reported.

Surface functionalization has been used for MgO NPs,^53^ which should have surface chemistry similar to Mg NPs encapsulated by a native oxide layer. For instance, variations in magnesium acetate and PVP concentrations for synthesis of MgO in ethylene glycol enabled selection between MgO NPs and nanowires.^54^ PEG, SDS, and CTAB^55^ have been used to form MgO nanoplates using sol–gel and hydrothermal methods for reactions between Mg(NO_3_)_2_ and NaOH.

Here, we synthesize Mg NPs in the presence of common capping agents, namely, PVP, PEG, SDS, and CTAB. We report the effects of capping agents on NP growth, their binding to the NP surface, and how their presence modifies the colloidal stability of Mg NPs. We then examine the effects of varying reaction parameters (reaction time, temperature, reducing agent, and Mg precursor) used in the syntheses in the presence of PVP.

Our results indicate that synthesis in the presence of PVP changes the Mg NP growth patterns, leading to reduced aggregation and improved colloidal stability. These effects are particularly striking in syntheses using Grignard reagents as precursors, where the structures obtained with PVP are well-defined, sharp NPs in contrast with the fused aggregates that are produced without a capping agent. Our findings are thus unlocking applications requiring well-dispersed, colloidally stable Mg nanostructures as well as paving the way for Mg NP syntheses using alternative and commonly available precursors.

## Materials and Methods

### Synthesis

Mg NPs were synthesized via reduction of an organomagnesium precursor, MgBu_2_, by a lithium arene complex, lithium naphthalenide, as previously reported.^30,33^ Chemicals were sourced from Sigma-Aldrich and used as received. All synthetic and purification steps described here were carried out in an Ar atmosphere, either in a glovebox (O_2_ and H_2_O concentrations below 5 ppm) or on a Schlenk line. The reducing agent was generated by sonicating lithium pellets (99%, 0.028 g, 4.0 mmol) and naphthalene (99%, 0.530 g, 4.1 mmol) in THF (99.9%, 10.75 mL) for 1 h to form a green lithium naphthalenide solution. MgBu_2_ (1.75 mL, 1.0 M, 1.75 mmol) was injected into the lithium naphthalenide solution under stirring, and the reaction was allowed to proceed for ∼20 h or less as indicated for kinetic experiments. The reaction was terminated by the addition of isopropanol (IPA, 99.5%, 6.25 mL) to deactivate the residual reducing agent and precursor, leaving a gray suspension of Mg NPs. The resulting NPs were purified by centrifugation and redispersion, twice in THF and three times in IPA before being redispersed in IPA for storage.

The following modifications to the scheme are made for further experiments. Capping agents were added to the lithium arene solution prior to sonication to ensure dissolution. The capping agents used were poly(vinylpyrrolidone) (PVP, *m* = 0.020 g, *M*_w_ = 10,000, 55,000, and 360,000 g mol^–1^; 2, 0.4, and 0.06 μmol, respectively), poly(ethylene glycol) (PEG, 0.048 g, *M*_w_ = 6000 g mol^–1^, 8 μmol), CTAB (99%, 0.064 g, 0.18 mmol), and SDS (99%, 0.050 g, 0.17 mmol). Reactions at 40 and 60 °C involved the heating of the lithium naphthalenide solution in an oil bath under stirring for 30 min before the MgBu_2_ injection. The reaction at 60 °C was performed under reflux to avoid solvent evaporation. Reactions at 0 °C were cooled in an ice bath. A further modification involves the use of other arenes for generation of the reducing agent, namely, biphenyl (99.5%, 0.638 g, 4.1 mmol) and phenanthrene (98%, 0.737 g, 4.1 mmol), which form blue and green complexes with Li, respectively. The final set of experiments involved substitution of the precursor for Grignard reagents, butylmagnesium chloride (BuMgCl) (2.0 M, 0.88 mL) and phenylmagnesium chloride (PhMgCl) (2.0 M, 0.88 mL). Since aggregation appears more extensive in the absence of a capping agent and given the faster kinetics, an increased amount of *M*_w_ = 10,000 PVP (0.120 g) was used with these precursors.

### Characterization

Samples were drop-cast from suspensions in IPA onto Si wafers for scanning electron microscopy (SEM) imaging, performed on a Quanta 650F FEG SEM operated at 5 kV and equipped with an Everhart–Thornley secondary electron detector. NPs were considered hexagonal platelets if they were visibly in the shape of a regular hexagon; their size was measured as the tip-to-tip distance. If one dimension was elongated, NPs were considered rod-like and size was measured as the elongated dimension. The numbers of observed and measured hexagons and rods are reported as *N*_hex_ and *N*_rod_, respectively. Since not all NPs lay flat on the substrate, sizes may be underestimated; however, this is likely an error equivalent across all samples, such that trends remain valid.

Inductively coupled plasma mass spectrometry (ICP-MS) was performed by using a PerkinElmer NexION 2000S mass spectrometer. Samples were digested in an aqueous matrix with 10% v/v of ultrapure nitric acid (max 10 ppt metal traces) for at least 10 min before analysis. Extinction spectra and experiments tracking the degree of NP suspension over time were measured using a Thermo Scientific Evolution 220 spectrophotometer, in which the beam measures extinction at approximately 1/3 of the solution height in a standard 1 cm path-length cuvette. Infrared spectra were acquired with a Thermo Scientific Nicolet iS 5 spectrometer using an attenuated total reflectance (ATR) attachment–sample preparation involved drying the product Mg NPs suspended in IPA (i.e., following purification as described above) at 120 °C, and background spectra were acquired before analyzing Mg powders.

X-ray photoelectron spectroscopy (XPS) data were collected at the Photoemission RTP, University of Warwick, using a Kratos Axis Ultra DLD spectrometer operated at room temperature with a monochromated Al Kα source. All samples were dried to powder by vacuum and stored in Ar at room temperature and only exposed to air for a few minutes during the mounting process. Additional experimental details of XPS are reported in the SI.

## Results and Discussion

### Capping Agents' Effects on NP Growth

Mg NPs were synthesized at room temperature through reduction of MgBu_2_ with an excess of freshly prepared lithium naphthalenide in an Ar atmosphere, yielding a mixture of hexagonal platelets and twinned rod-like NPs with a constant aspect ratio (as described previously^30,33−35^). To assess the effect of capping agents, the reactions were run with and without a 10:1 (with respect to monomer unit for polymers) ratio of a Mg precursor to either PVP (*M*_w_ = 10,000, 55,000, and 360,000), PEG (*M*_w_ = 6,000), CTAB, and SDS. This range of capping agents was chosen to span charge states, from cationic (CTAB) to neutral (PEG, PVP) and anionic (SDS).

**Figure 1:**
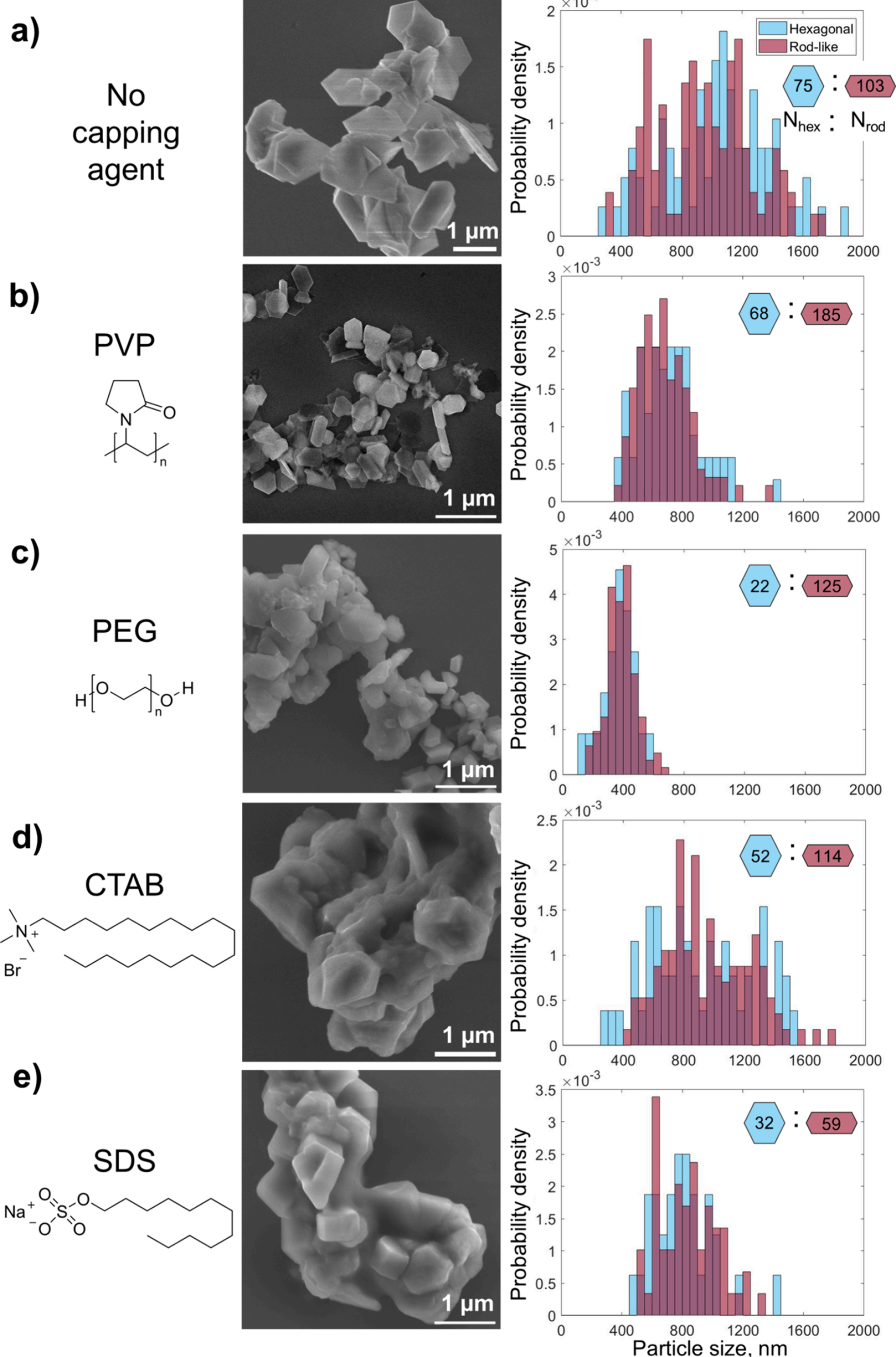
Capping agent effects on Mg NPs obtained from the reduction of MgBu_2_. Molecular schematic of capping agents, SEM images, and size distribution histograms for (a) bare Mg NPs and Mg NPs synthesized in the presence of (b) PVP *M*_w_ = 10,000, (c) PEG, (d) CTAB, and (e) SDS. Blue bars show the size distribution of hexagonal platelets, and red bars show the size distribution of rod-like structures, both measured tip to tip. Average NP size and standard deviation for hexagonal and rod-like NPs are reported in Table S1.

The reactions yielded Mg NPs with a strikingly different extent of aggregation. In most instances, the NPs are smaller in the presence of capping agents when compared to “bare” reactions containing only the reducing agent and Mg precursor (Figure 1, Figures S1 and S2, and Table S1). The mean size and standard deviation for bare Mg NPs are 1030 ± 340 for hexagonal platelets and 960 ± 310 nm for rod-like structures (Figure 1a and Figure S1a). NP size decreases while retaining a similar relative standard deviation in the presence of most capping agents, with 720 ± 200 hexagonal platelets and 690 ± 170 nm rod-like with PVP *M*_w_ = 10,000 (Figure 1b and Figure S1b), 310 ± 100 hexagonal platelets and 360 ± 130 nm rod-like with PEG (Figure 1c and Figure S1e), and 800 ± 200 hexagonal platelets and 840 ± 190 nm rod-like with SDS (Figure 1e and Figure S1g). NPs produced in the presence of CTAB (900 ± 330 hexagonal platelets and 950 ± 280 nm rod-like, Figure 1d and Figure S1f) are not of markedly different size than those synthesized without capping agents.

Capping agents also affect the yield of NPs, as measured by ICP-MS. The yield obtained in the presence of PVP *M*_w_ = 10,000 was the highest (27%), followed by bare Mg (23%), SDS (22%), PEG (19%), and CTAB (13%) (Figure S2). The capping agents thus alter the reaction yield by a factor of ∼2, with PVP promoting reduction and CTAB decreasing it. Considering these yields together with NP size distribution, assuming the constant aspect ratio,^30^ provides insight on the effect of capping agents on nucleation and growth in the studied reactions. For instance, PVP-containing reactions produced smaller NPs and a higher yield than other reactions, indicating that more nuclei are formed, while growth could be reduced by the surface-stabilizing effects of this effective capping ligand. Similar size distribution and a notable decrease in the reaction yield are observed with CTAB compared to bare NPs, indicating suppressed nucleation. Lastly, the addition of SDS or PEG did not significantly modify the yield compared to capping agent-free reactions but resulted in lower average NP size, suggesting the promotion of nucleation and possible reduction of growth.^47,48^

### Surface Binding of Capping Agents

Strongly binding capping agents are key to improving the colloidal stability and can provide pathways for further functionalization. XPS confirms the elements and electronic states present at the surface and allows for an understanding of surface chemistry and binding. Specifically, wide energy range scans reveal the presence of Mg and O from the NPs, F from contamination from the PTFE-based vacuum grease, and C from the capping ligands and magnesium carbonate; for the PVP-containing Mg NPs, N is also observed.

The Mg 2p region for NPs produced with the capping agents PVP *M*_w_ = 10,000, PEG, CTAB, and SDS was fitted with 4 Gaussians corresponding to MgCO_3_, MgO, Mg(OH)_2_, and Mg metal (positions reported in Table S2). MgO and Mg(OH)_2_ are expected due to Mg’s reactivity with atmospheric oxygen and moisture, while the carbonate arises from adsorption of CO_2_ and subsequent reaction with surface hydroxide groups.^56^ The observation of a Mg metal signal implies that the oxide/hydroxide layer is thinner than the sampling depth of XPS, i.e., less than ∼10 nm. In addition to their Mg 2p contributions, the presence of Mg oxide, hydroxide, and carbonate is confirmed through their appearance in the O 1s region (Figure S3). Note that a further signal arises from organic O from PEG or possibly from remaining solvents.

The C 1s region indicates, as expected for solvents, C–O signals as well as C=O, O=C–O, and carbonate indicative of CO_2_ binding, further providing consistency to the assignments made for the other elements. Additionally, C 1s regions for all capping ligands show the presence of C–F due to PTFE-based vacuum grease not being removed on purification. This is further evidenced by the F 1s region (Figure S3 and Table S2).

**Figure 2:**
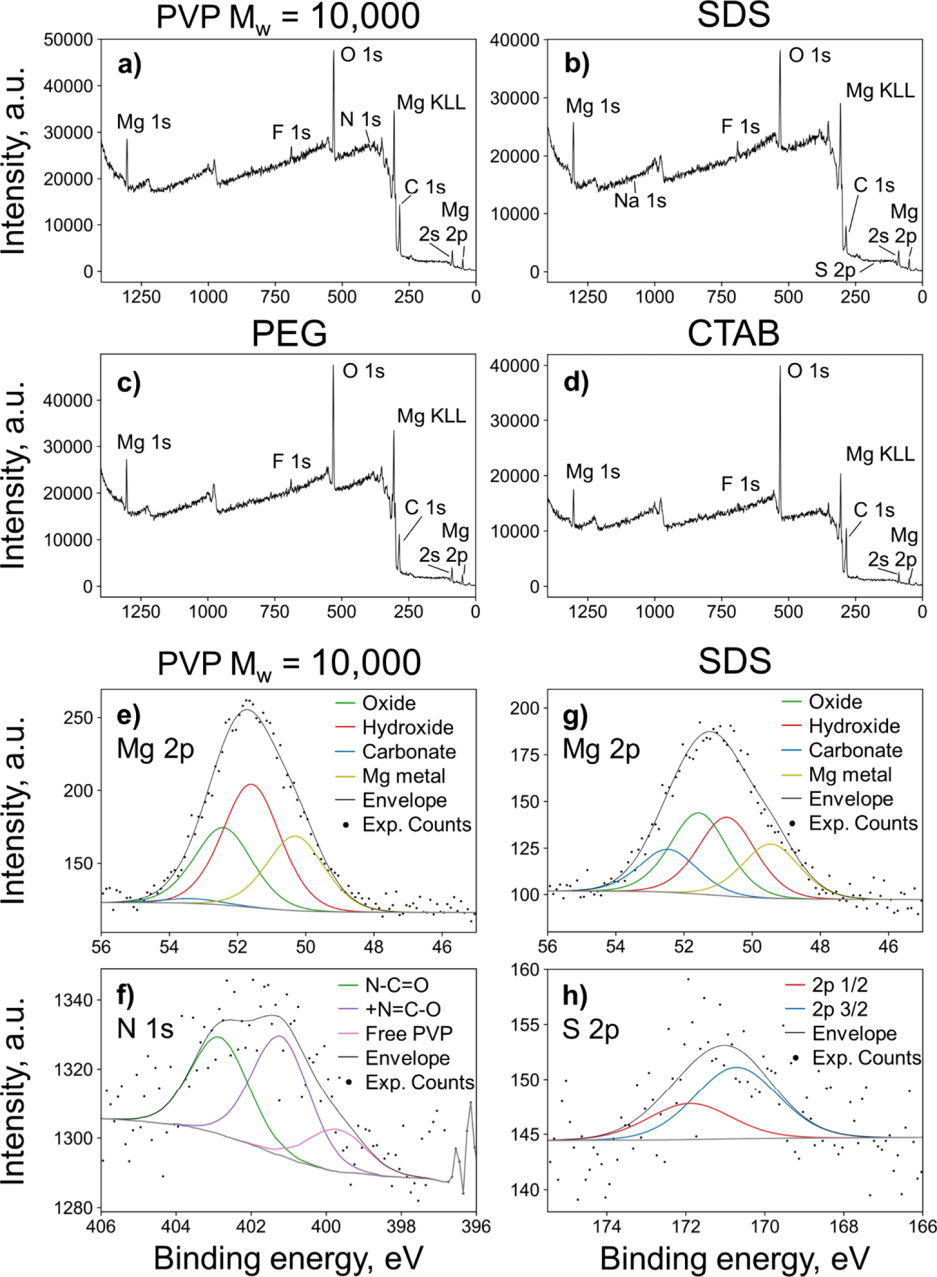
Capping agents on purified, dried Mg NPs. XPS survey spectra of Mg NPs synthesized in the presence of (a) PVP *M*_w_ = 10,000, (b) SDS, (c) PEG, and (d) CTAB. High-resolution XPS spectra of the (e) Mg 2p and (g) N 1s regions for Mg NPs with PVP *M*_w_ = 10,000, and the (g) Mg 2p and (h) S 2p regions for Mg NPs with SDS. Additional data reported in Figure S3.

XPS indicates that PVP binds to the surface of the NPs, consistent with the significant improvement observed in aggregation and colloidal stability. For PVP, the C 1s region contains an additional peak arising from N–C=O moieties, indicating this capping ligand’s presence on the NP surface. This is supported by the N 1s region (Figure 2f), which can be fit with three peaks - free PVP (399.7 eV),^57,58^ N–C=O (401.2 eV), and ^+^N=C–O^–^ (402.9 eV). Additional N 1s peaks at higher binding energies imply that electron density is being donated away from N. Thus, some pyrrolidone moieties along the PVP backbone appear to be bound to the NP surface, but not all, as expected for a long-chain polymer. Interestingly, Mg 2p peaks are shifted to higher binding energies (by ∼1 eV) for Mg with PVP *M*_w_ = 10,000 compared to those from other capping agents, suggesting that electron density on Mg is reduced. PVP also appears to suppress carbonate formation (Figure 2e), compared to other capping agents (SDS in Figure 2g, others in Figure S3), also consistent with surface binding.

The XPS data are less conclusive for other capping agents. The presence, and probable binding, of SDS can be confirmed by peaks in the S 2p and Na 1s regions (Figure S3 and Table S2). PEG’s C and O signals overlap with crowded spectral regions, providing little indication of binding (Table S2). Lastly, CTAB appears to have been removed to below the limit of detection upon purification, signature of poor to no binding. Indeed, neither Br 3d (∼69 eV) nor N 1s (∼400 eV) was observed in the survey spectrum of (purified) Mg synthesized in the presence of CTAB.

**Figure 3:**
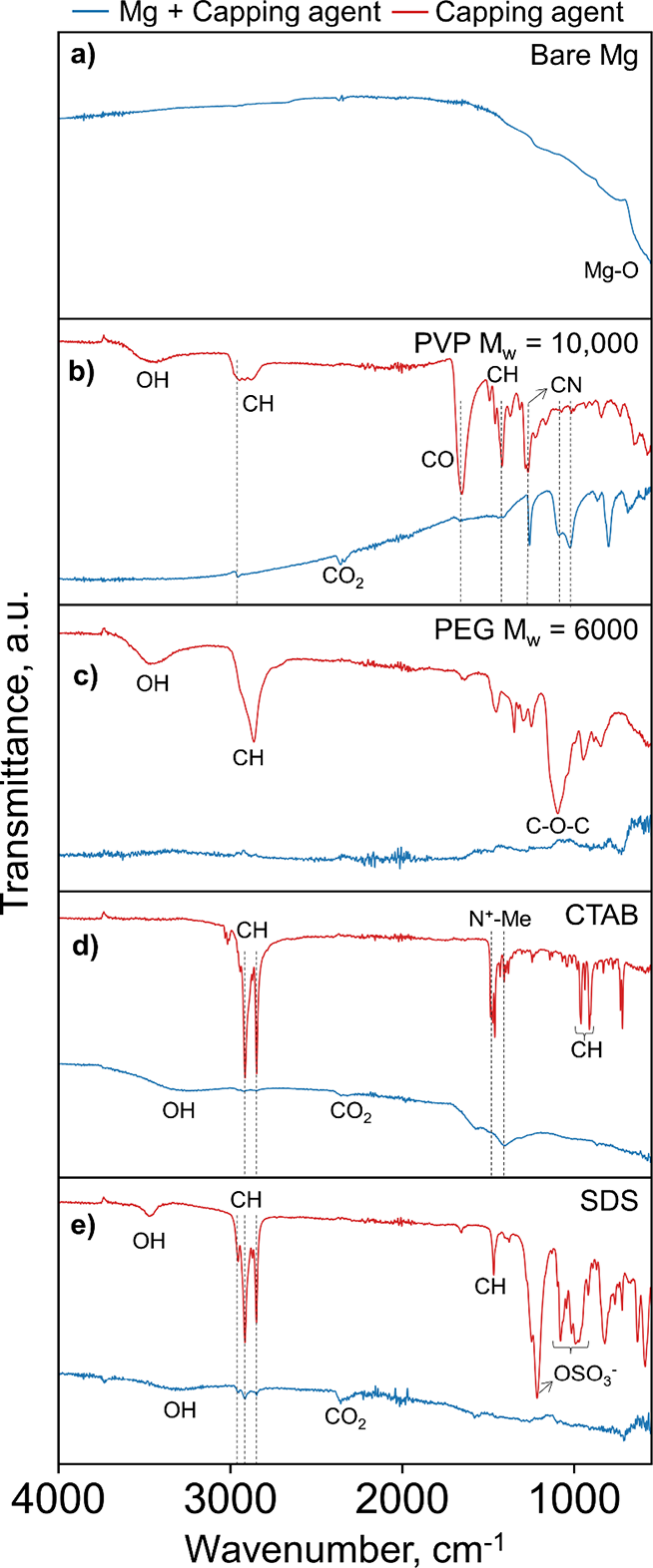
Capping agents and purified dried Mg NPs. IR spectra of neat capping agents (red traces) and Mg NPs (blue traces) synthesized (a) without capping agent and in the presence of (b) PVP *M*_w_ = 10,000, (c) PEG, (d) CTAB, and (e) SDS.

IR spectroscopy of NPs centrifuged and redispersed multiple times (see Materials and Methods) to remove nonspecifically bound capping agents confirms that PVP binds firmly to Mg NPs. The IR spectrum of bare Mg NP is shown in Figure 3a, with Mg–O–Mg modes from MgO as a band spanning 700–550 cm^–1^; the expected O–H absorption^59^ at 4000–3000 cm^–1^ due to adsorbed water and/or Mg(OH)_2_ was weak and obscured by the baseline signal. Meanwhile, the band at 700–1500 cm^–1^ is likely from the O–H and C–H bending modes arising from small amounts of surface contamination. The IR spectrum of Mg NPs synthesized in the presence of PVP displays additional bands indicative of PVP (Figure 3b), notably a broad peak at ∼2900 cm^–1^ due to C–H symmetric and asymmetric stretching modes, a C=O stretch from the pyrrolidone ring at 1652 cm^–1^, C–H bends at 1419 cm^–1^, and C–N stretches at 1268, 1015, and 1001 cm^–1^.^58^ The spectrum also contains a broad peak around 3450 cm^–1^ that arises from surface O–H.^58^

Meanwhile, none of the characteristic PEG peaks are observed (Figure 3c), such as the broad peaks for O–H at 3465 cm^–1^ from terminal O–H of the PEG chains, C–H stretches at 2863 cm^–1^, and C–O–C asymmetric stretching at 1095 cm^–1^.^60^ This suggests the absence of PEG after purification, which is consistent with the XPS results. Similarly, CTAB and SDS do not feature prominently in the IR spectra of the purified Mg NPs. CTAB modes are expected as follows (Figure 3d): H–C–H vibrations at 2915 and 2847 cm^–1^, N^+^–Me symmetric stretches around 1475 cm^–1^, and out-of-plane C–H stretches at 960 and 911 cm^–1^.^61^ Only the H–C–H modes are repeated in the spectrum of Mg NPs synthesized in the presence of CTAB, suggesting that this may be contamination. Finally, the only peaks in common between the Mg NPs synthesized with SDS and neat SDS spectra (Figure 3e) are C–H stretching peaks at 2955, 2915, and 2849 cm^–1^. None of the SDS’s prominent characteristic peaks, including a H–C–H bend at 1468 cm^–1^, the asymmetric sulfate, −OSO_3_^–^, stretching peak at 1215 cm^–1^, and a symmetric sulfate stretching band from 1100 to 900 cm^–1^,^62^ appear in the spectrum of Mg NPs, confirming the poor binding suggested by XPS.

### Colloidal Stability

Capping agents play an important role, via steric or electrostatic repulsion, in controlling the aggregation of NPs during and after synthesis, affecting colloidal stability. SEM images (Figure 1 and Figure S1) suggest that NPs synthesized in the presence of CTAB and SDS are significantly aggregated immediately postsynthesis. The qualitative dispersion of NPs improves greatly from bare NPs to those synthesized with PEG and furthermore for PVP. PVP-containing NPs are well separated with clearly discernible facets; these have shapes consistent with the single-crystalline and singly twinned structures reported previously.^34^ However, drying artifacts are possible, can depend on the capping ligand, and prevent quantitative conclusions; we prepared the samples in an identical manner to minimize drying variations that could be due to preparation.

**Figure 4:**
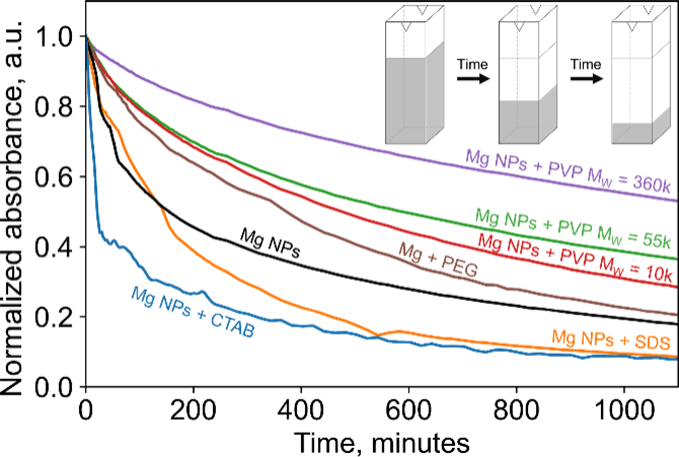
Sedimentation of Mg NPs suspended in IPA. Absorbance at 450 nm as a function of time for bare Mg NPs and NPs synthesized in the presence of the capping agents PVP (*M*_w_ = 10,000, 55,000, and 360,000), PEG, CTAB, and SDS, as indicated on the plot. Absorbance at 600 and 750 nm is reported in Figure S5.

Tracking sedimentation reveals large variations in colloidal stability between the products obtained in the presence of different capping agents with PVP outperforming others in terms of colloidal stability. Because of their LSPRs, metallic Mg NPs have a strong (Figure S4) absorbance profile through which intensity relays concentration and thus degree of sedimentation (Figure 4 and Figures S5 and S6). Absorbance at 450 nm (Figure 4) and at 600 and 750 nm (Figure S5) indicates that adding PVP improves colloidal stability compared to bare Mg NPs. Here, we studied Mg synthesized in the presence of three different PVP *M*_w_, 10,000, 55,000, and 360,000 g mol^–1^, for which binding is expected to be identical. Of the molecular weights used, 360,000 improved colloidal stability the most, possibly because larger *M*_w_ can form thicker shells, but led to more difficult postsynthetic purification. Thus, PVP *M*_w_ = 10,000 was used in further experiments. Meanwhile, NPs synthesized in the presence of SDS and CTAB sedimented rapidly, likely due to both the aggregated state of the as-synthesized product and further aggregation in solution as these capping agents do not bind to the NP’s surface. Specifically, after 19 h, the 450 nm absorbance of Mg NPs synthesized in the presence of SDS and CTAB both decreased to around 8% of their initial absorbance, while it reached 29, 36, and 53% for PVP of *M*_w_ = 10,000, 55,000, and 360,000, respectively (Table S4 for other wavelengths). Furthermore, CTAB’s effect on short-term stability is particularly poor, evidenced by the 50% decrease in absorbance observed after only 22 min (Table S4). No degradation or other irreversible changes were observed in the time scale of the experiment, as confirmed by the similar shape of the absorbance spectrum for samples before sedimentation and after redispersion by sonication (Figure S4).

To determine whether aggregation played a role in colloidal stability measurements, time-resolved full extinction spectra were acquired for Mg NPs synthesized with PVP *M*_w_ = 10,000 (Figure S6). Significant aggregation would lead to changes in spectral features that are not observed, confirming that variations in aggregation are minimal during sedimentation.

Lastly, the metallic nature of the Mg core was confirmed by acquiring low-loss EELS and mapping the bulk metallic plasmon signal at ∼10.6 eV (Figure S7). These NPs thus retain a metallic core when synthesized in the presence of PVP, as expected.

### PVP Enables the Use of Grignard Precursors

**Figure 5:**
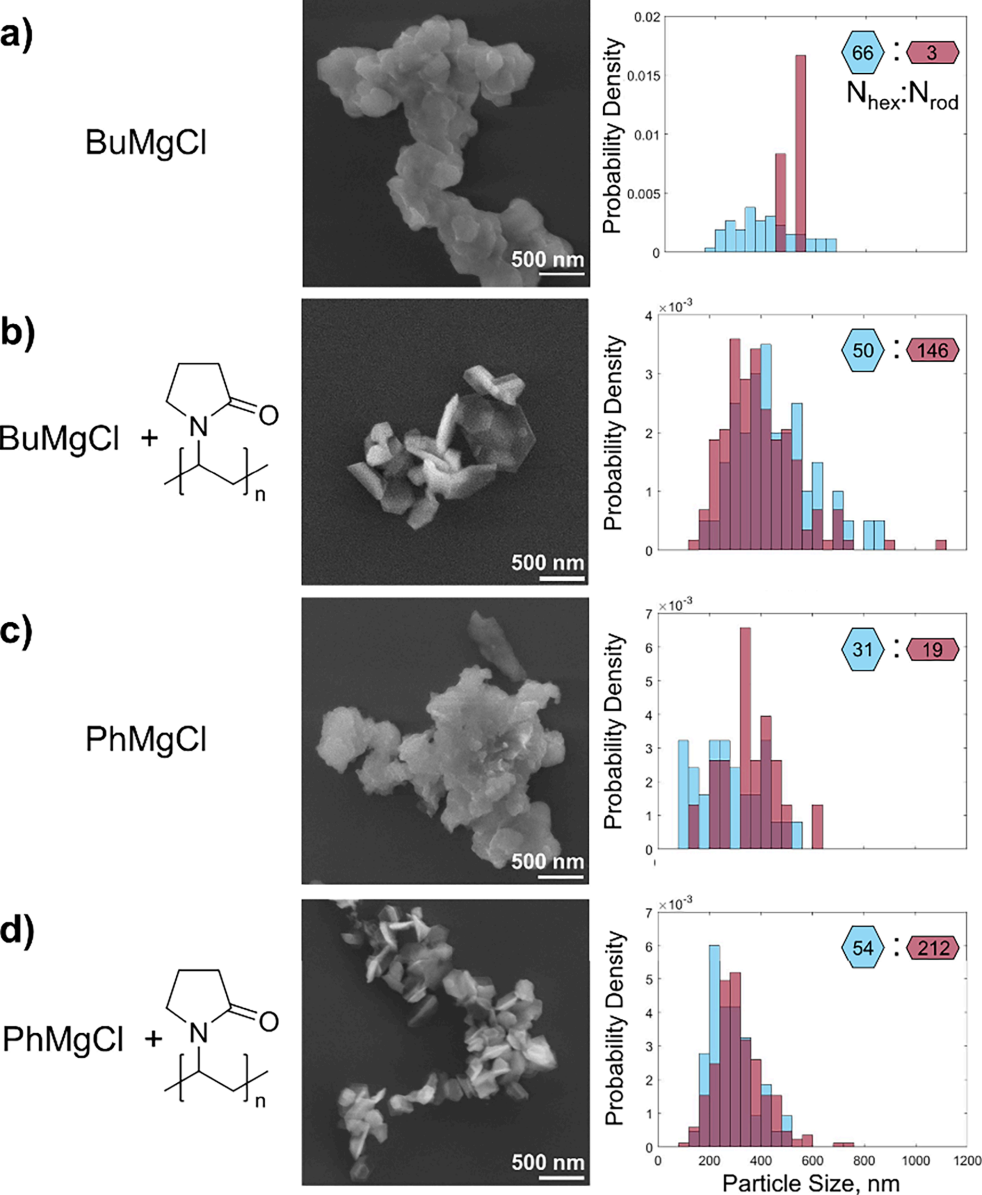
Mg NP syntheses with Grignard reagents improved by the addition of PVP *M*_w_ = 10,000. SEM images and NP size distributions for syntheses with (a) BuMgCl without and (b) with PVP; PhMgCl (c) without and (d) with PVP. Additional SEM images and average hexagonal and rod-like NP sizes are reported in Figure S8 and Table S5, respectively.

While most Grignard (RMgX) reagents can be reduced by lithium naphthalenide to Mg metal, the capping agent-free reduction of BuMgCl (Figure 5a and Figure S8a) and PhMgCl (Figure 5c and Figure S8c) leads to aggregated, fused NPs. These structures are indicative of aggregation during synthesis, followed by further growth via deposition of Mg on the aggregates. The obtained products are therefore of little use, as they consist of large heterogeneous structures that rapidly sediment.

The level of aggregation of Mg NPs from BuMgCl and PhMgCl was significantly improved by the presence of PVP during the synthesis. Preliminary experiments determined that the reduction rate is significantly higher for BuMgCl and PhMgCl compared with MgBu_2_. Within 5 min of reaction, Mg yield for BuMgCl and PhMgCl is 15 and 20%, respectively, compared to 1.2% after 15 min for MgBu_2_ in the presence of PVP (Figure S9). To stabilize this faster reaction, an increased ratio of the 6:10 PVP monomer:Mg precursor was used, which yielded faceted, discrete NPs with clearly identifiable single-crystalline and singly twinned (rod-like) morphologies (Figure 5 and Figure S8). For BuMgCl, hexagonal platelets of 450 ± 150 and rod-like NPs of 400 ± 140 nm were obtained; the reduction of PhMgCl yielded hexagonal platelets of 280 ± 80 and rod-like NPs of 320 ± 100 nm. These sizes are consistent with the estimated size, from the outermost NPs in the fused aggregates, obtained in the PVP-free reduction of BuMgCl and smaller than those of the NPs obtained with PVP-free PhMgCl (Table S5).

Importantly, the NPs obtained from Grignard reagents in the presence of PVP are remarkably well-dispersed, indicating that growth occurs mainly on individual NPs (as opposed to on aggregates). This result highlights PVP’s ability to interact with the surface of Mg NPs, preventing aggregation during the synthesis. Furthermore, this finding suggests that widely available Grignard reagents can be used to produce well-dispersed Mg NPs at substantially reduced reaction times (minutes vs hours) for applications in nanomedicine, sensing, and nanoplasmonics technologies.

### Reaction Time, Temperature, and Electron Carrier Effects in the Presence of PVP

Owing to the promise shown by PVP as a capping agent for Mg NPs, we carried out further systematic investigations of PVP-containing reductions of MgBu_2_. Here, we overview the effect of reaction time, temperature, and electron carrier on the size distribution of Mg NPs obtained in the presence of PVP, with full details discussed in the SI.

We reported above (Figure 1) that PVP reduced the average NP size compared with PVP-free syntheses. However, we note that the NPs obtained here are larger than most bare NPs reported previously,^30^ an apparent inconsistency. We tentatively attribute this difference to the presence of an AlEt_3_ additive (a viscosity reducing agent) in previous batches of MgBu_2_.^30^ Such metal salt additives have been demonstrated to reduce NP size.^30^ Based on the information available from the supplier (Sigma), MgBu_2_ no longer contains AlEt_3_ and we have used this additive-free precursor for all syntheses in this paper.

Kinetics data, including size (Figures S9–S11 and Table S6) and yield (Figure S9) as a function of reaction time, indicate that nucleation occurs throughout the reaction. In the presence of PVP, the average NP size (Table S6) increases from 240 ± 45 for hexagonal platelets and 240 ± 70 nm for rod-like NPs after 15 min, to 720 ± 200 and 690 ± 170 nm after ∼20.5 h (Figures S9 and S10). NP growth occurs rapidly in the first 3 h (580 ± 170 hexagonal platelets and 660 ± 160 nm rod-like), while the yield of Mg (Figure S9) continues to increase until ∼6 h, suggesting continuous nucleation and growth rather than nucleation only in the early stages of the reaction. As discussed above, PVP appears to promote nucleation due to the reduced size and increased yield with respect to capping agent-free syntheses. Additionally, growth rate can be slowed since PVP binds to the surface,^63,64^ which would result in a reduced NP size under a continuous nucleation regime. Ostwald^65^ and digestive ripening,^66^ which would lead to an increase and decrease in polydispersity with time, respectively, are not observed in this system: Increasing the reaction time from 3 to ∼20.5 h causes little change in polydispersity, from 30 and 24% for hexagonal platelets and rod-like NPs, respectively, at 3 h to 28 and 25% at 20.5 h.

Changing the reaction temperature affects the production of NPs, by changing the nucleation and growth rates. Mg NPs were synthesized at temperatures varying from 0 to 60 °C (limited by THF’s boiling point of 66 °C, Figures S12 and S13) for ∼20.5 h total; this ensured the completion of the reduction as the maximum yield is reached at room temperature after 6 h (Figure S9) when reducing MgBu_2_ with lithium naphthalenide in the presence of PVP at room temperature. Yield increased almost linearly with temperature, giving 11, 27, 34, and 44% at 0, 20, 40, and 60 °C, respectively (Figure S12). However, the final NP size with PVP did not vary significantly with temperature within the range studied (Table S7), implying less nucleation at lower temperatures.

**Figure 6:**
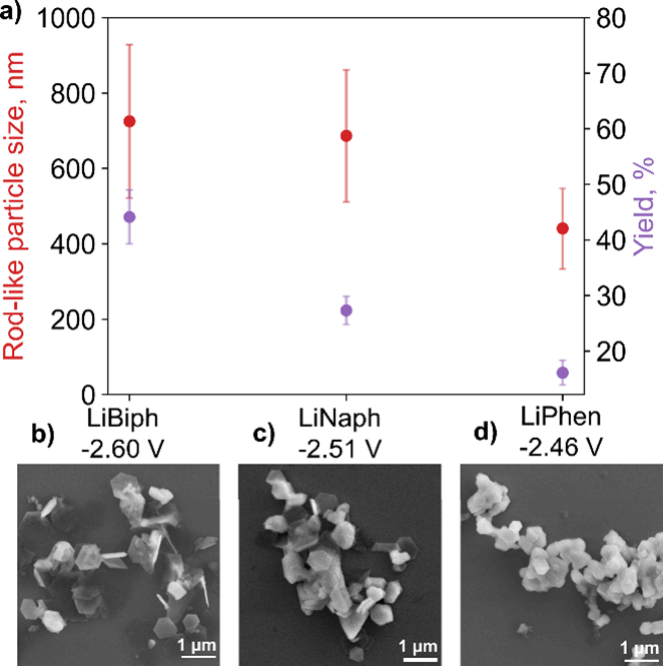
Effect of electron carrier on rod-like NP formation. (a) Comparison of rod-like NP size (red) and NP yield (purple) for NPs synthesized with different electron carriers in the presence of PVP *M*_W_ = 10,000 for ∼20.5 h and SEM images of NPs formed from reactions using (b) biphenyl (LiBiph), (c) naphthalene (LiNaph), and (d) phenanthrene (LiPhen). Error bars show the standard deviation of the NP distributions (red) or that of three yield measurements (purple). Additional SEM images and average hexagonal and rod-like NP sizes are reported in Figure S14 and Table S8, respectively.

Using other lithium arenides also leads to changes in the yield and average NP size. NPs synthesized using lithium biphenylide (hexagonal platelets of 800 ± 210 and rod-like NPs of 720 ± 200 nm, Figure 6, Figures S13 and S14, and Table S8) as a reducing agent are comparable in average size to those from the weaker lithium naphthalenide (hexagonal platelets of 720 ± 200 and rod-like NPs of 690 ± 170 nm Figure 6 and Table S8). However, the yield obtained with lithium biphenylide is significantly higher (44 vs 27%), indicating an increase in nucleation and growth for the stronger reducing agent. Meanwhile, both the average size (hexagonal platelets of 400 ± 100 and rod-like NPs of 440 ± 110 nm, Figures S13–14, Table S8) and the yield (16%) decrease when using lithium phenanthride. Additionally, NPs appear less faceted and more aggregated with lithium phenanthride. Assuming a constant NP aspect ratio (as demonstrated in previous syntheses^30^), the size decrease leads to an NP volume decrease by up to a factor of 8, while the yield decreases only two- to threefold compared to the other reducing agents. There are therefore more and much smaller NPs in the lithium phenanthride reaction, indicating inhibited growth.

## Conclusions

The effects of common capping agents on the synthesis and colloidal stability of metallic Mg NPs were assessed. We found that NPs synthesized in the presence of PVP show reduced aggregation and postsynthetic sedimentation. XPS and FTIR analyses confirmed the presence and binding of PVP *M*_w_ = 10,000. Other capping agents such as CTAB, PEG, and SDS performed poorly at colloidally stabilizing the NPs, and their weak or nonexistent signal in purified samples suggests little to no binding to the MgO surface.

In addition to improving the colloidal stability, the presence of PVP during Mg NP synthesis enabled well-separated NPs from a wide range of precursors. Here, we demonstrated the use of reactive BuMgCl and PhMgCl and we anticipate that this approach can be extended to most Grignard reagents. Finally, we surveyed the effects of various reaction parameters on the average NP size and reaction yield for the reduction of MgBu_2_ in the presence of PVP. These results provide synthetic routes that generate colloidally stable and well-dispersed Mg NPs for plasmonic and other applications.

## Supporting Information Available

Details of XPS measurements, additional SEM images, average NP size data and histograms, reaction yield results, extinction spectra, additional time-resolved extinction spectra, XPS fits, and compositional analysis from XPS (PDF)
