# Supplementary material for: Capping Agents Enable Well-Dispersed and Colloidally Stable Metallic Magnesium Nanoparticles
Source: J Phys Chem C Nanomater Interfaces. 2024 Mar 12;128(11):4666–76. doi: 10.1021/acs.jpcc.4c00366 (PMC10961833; doi:10.1021/acs.jpcc.4c00366)
Supplement: Supplementary file 1 — jp4c00366_si_001.pdf [file jp4c00366_si_001.pdf]

Supporting Information for

Capping Agents Enable Well-Dispersed and Colloidally Stable Metallic Magnesium Nanoparticles

Thomas M. R. Wayman, Vladimir Lomonosov,* and Emilie Ringe*

Department of Materials Science and Metallurgy, University of Cambridge, 27 Charles Babbage Road, Cambridge CB3 0FS, United Kingdom and Department of Earth Sciences, University of Cambridge, Downing Street, Cambridge CB2 3EQ, United Kingdom

Additional XPS Experimental Details

The dried samples were deposited on electrically-conductive carbon tape, mounted on to a sample bar with a layer of filter paper between the samples and the sample bar to ensure electrical isolation and hence differential charging, before being loaded into a Kratos Axis Ultra DLD spectrometer which possesses a base pressure below 1 x 10^{-10} mbar.
XPS measurements were performed in the main analysis chamber, with the sample being illuminated using a monochromated Al Kα x-ray source (hν = 1486.7 eV). The measurements were conducted at room temperature and at a take-off angle of 90° with respect to the surface parallel. The core level spectra were recorded using a pass energy of 20 eV (resolution approx. 0.4 eV), from an analysis area of 300 mm x 700 mm . The work function and binding energy scale of the spectrometer were calibrated using the Fermi edge and $3d_{5/2}$ peak recorded from a polycrystalline Ag sample prior to the commencement of the experiments. To prevent surface charging the surface was flooded with a beam of low energy electrons from a charge neutraliser throughout the experiment and this necessitated recalibration of the binding energy scale. To achieve this, the C-C/C-H component of the C 1*s* spectrum was referenced to 285.0 eV. The data were analysed in the CasaXPS package using Shirley backgrounds and mixed Gaussian-Lorentzian (Voigt) lineshapes. For compositional analysis, the analyser transmission function has been determined using clean metallic foils to determine the detection efficiency across the full binding energy range. Mg signals were fit as single peaks as the spin-orbit splitting for Mg is less than instrumental resolution.

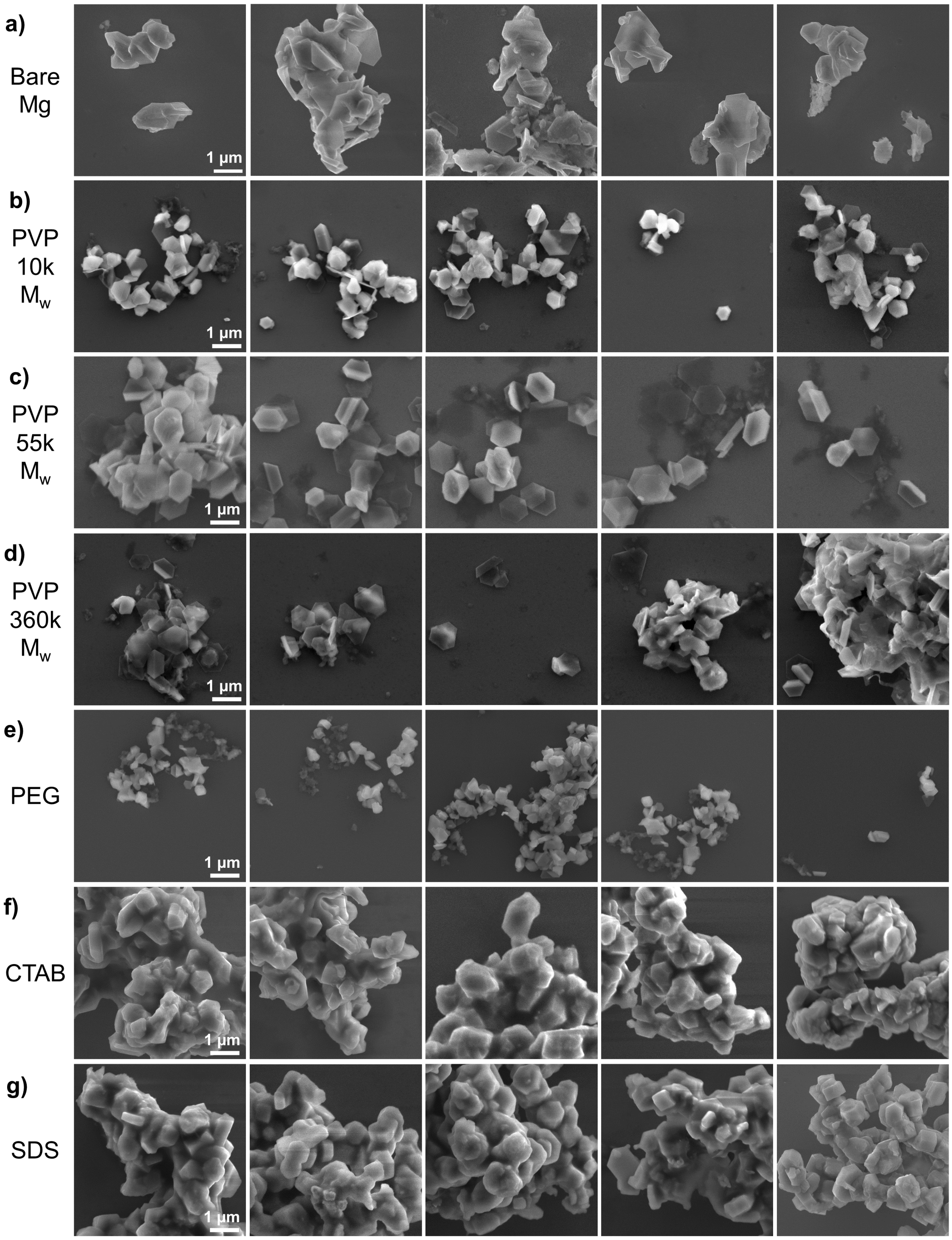

Figure S1. SEM images of (a) bare Mg NPs and Mg NPs synthesized in the presence of (b) PVP M_W = 10,000, (c) PVP M_W = 55,000, (d) PVP M_W = 360,000, (e) PEG, (f) CTAB, and (g) SDS.

Table S1. Average NP size, standard deviation and number of NPs counted for hexagonal platelet and rod-like Mg NPs synthesized in the presence of PVP, PEG, CTAB and SDS.

Capping agent	Average hexagonal platelet size, nm	Number of hexagonal platelets counted	Average rod-like particle size, nm	Number of rod-like particles counted
None	1030 ± 340	75	960 ± 310	103
PVP M_w = 10,000	720 ± 200	68	690 ± 170	185
PVP M_w = 55000	1100 ± 180	145	1100 ± 190	190
PVP M_w = 360000	920 ± 210	160	810 ± 170	240
CTAB	900 ± 330	52	950 ± 280	114
SDS	800 ± 200	32	840 ± 190	59
PEG	310 ± 100	36	360 ± 130	191

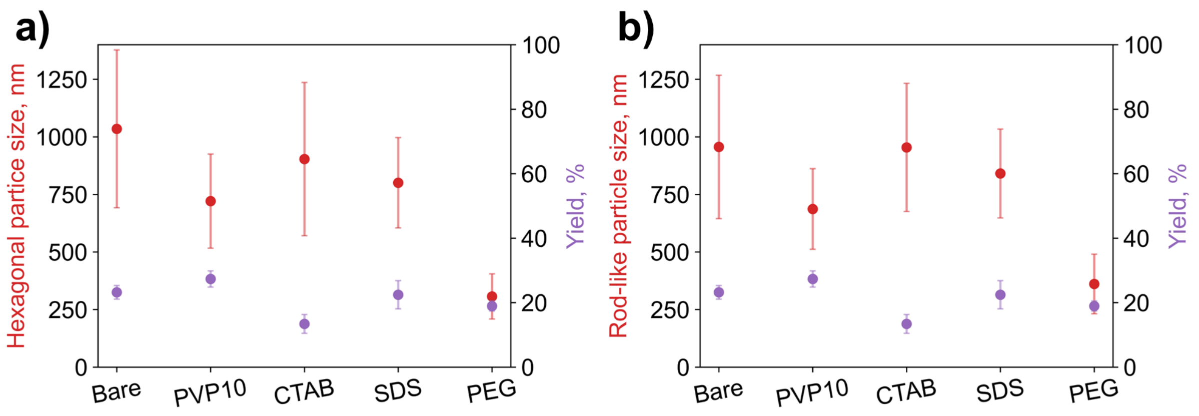

Figure S2. Average NP size (red markers, left axes) and reaction yield (purple markers, right axes) for (a) hexagonal platelets and (b) rod-like shapes for syntheses of Mg NPs without capping agent (Bare) and in the presence of PVP (M_w = 10,000, PVP10), CTAB, SDS and PEG. Error bars show standard deviation of particle distributions (red) or that of 3 yield measurements (purple).

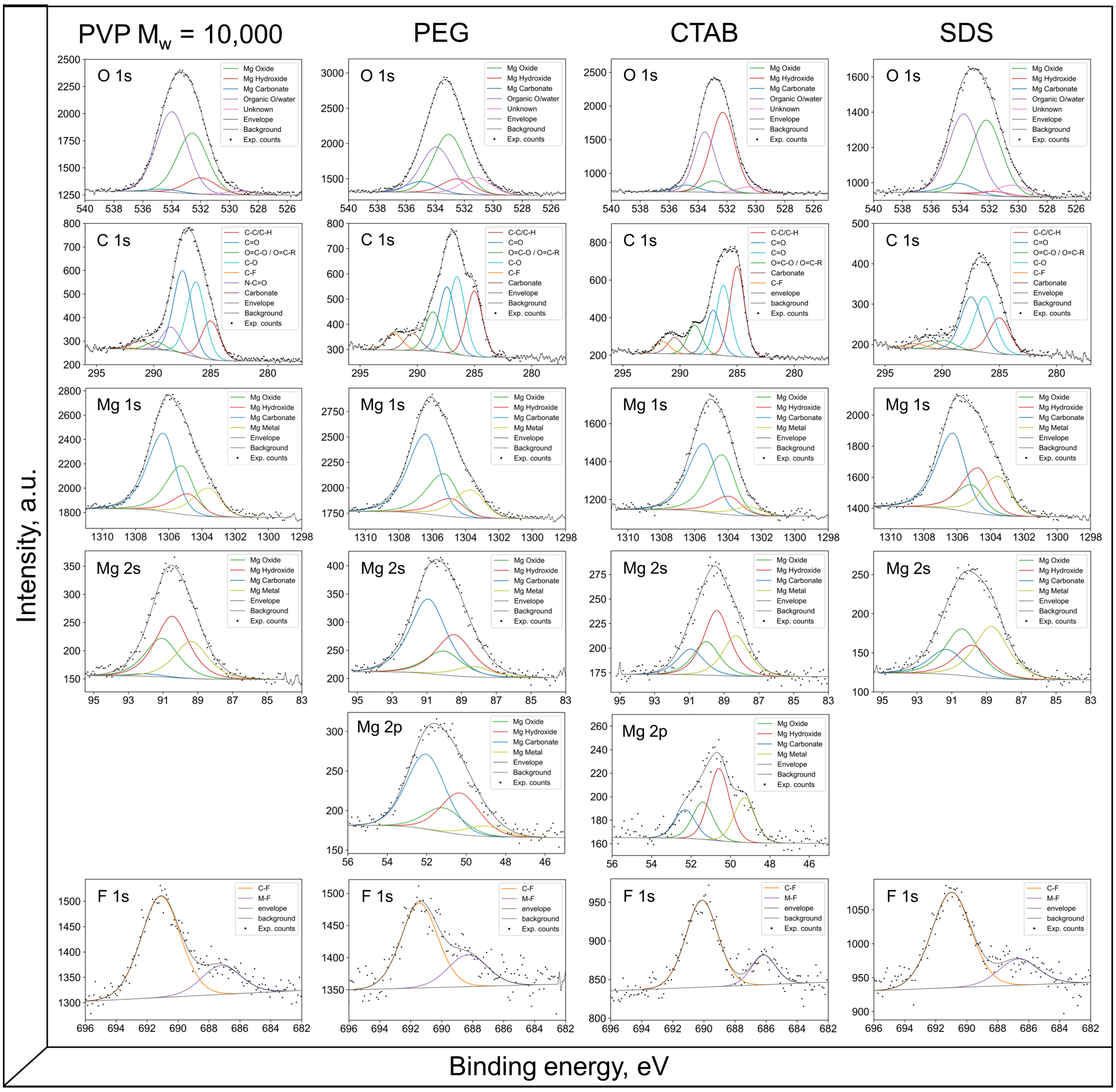

Figure S3. HRXPS of O 1s, C 1s, Mg 1s, Mg 2s and F 1s regions for Mg NPs synthesized in the presence of PVP M_w = 10,000, PEG, CTAB and SDS.

Table S2. Peak maxima of Gaussians, in eV, fitted in regions of interest scanned in HRXPS for the capping agents PVP M_w = 10,000, PEG, CTAB and SDS.

Parent region	Assigned Peak	PVP10k	PEG	CTAB	SDS
Mg1s	Mg Oxide	1305.3	1305.3	1304.4	1305.2
	Mg Hydroxide	1304.9	1305	1304.0	1304.8
	Mg Carbonate	1306.4	1306.4	1305.5	1306.2
	Mg Metal	1303.7	1303.7	1302.8	1303.5
Mg 2s	Mg Oxide	91.1	90.1	90.0	90.4
	Mg Hydroxide	90.4	89.4	89.4	89.9
	Mg Carbonate	92.1	90.9	90.9	91.3
	Mg Metal	89.3	88.4	88.3	88.7
Mg 2p	Mg Oxide	52.4	51.3	51.4	51.5
	Mg Hydroxide	51.6	50.4	50.6	50.7
	Mg Carbonate	53.4	52.1	52.3	52.5
	Mg Metal	50.3	49.1	49.3	49.4
O 1s	Mg Oxide	532.5	533.1	532.9	532.2
	Mg Hydroxide	532.0	532.5	532.3	539.6
	Mg Carbonate	534.7	535.0	534.8	534.2
	Organic O/water	534.0	533.9	533.5	533.7
	Organic O				
	Unknown	529.3	531.2	530.5	530.4
C 1s	C-C/C-H	285.0	285.0	285.0	285.0
	C=O	287.4	287.4	287.1	287.4
	O=C-O/O=C-R	289.8	288.6	288.7	289.8
	C-O	286.3	286.5	286.2	286.3
	C-F	295.8	292.0	291.5	292.4
	N-C=O	288.5			
	carbonate	291.1	290.4	290.5	291.1
F 1s	C-F	691.1	691.4	690.1	690.9
	M-F	687.1	688.3	686.2	686.6
	F				
N 1s	N-C=O	401.2			
	+N=C-O	402.9			
	PVP	399.7			
S 2p	2p 1/2				171.8
	2p 3/2				170.7
Na 1s	Na 1s				1073.6

Table S3. Surface elemental composition as determined by XPS.

	Elemental Composition (atomic %)						
Sample	Mg	F	O	C	N	S	Na
PVP 10k	15.5	5	33.5	44	2	-	-
PEG	17	3	42	38	-	-	-
CTAB	9	2	41	48	-	-	-
SDS	21.5	6	38	33.5	-	0.5	0.5

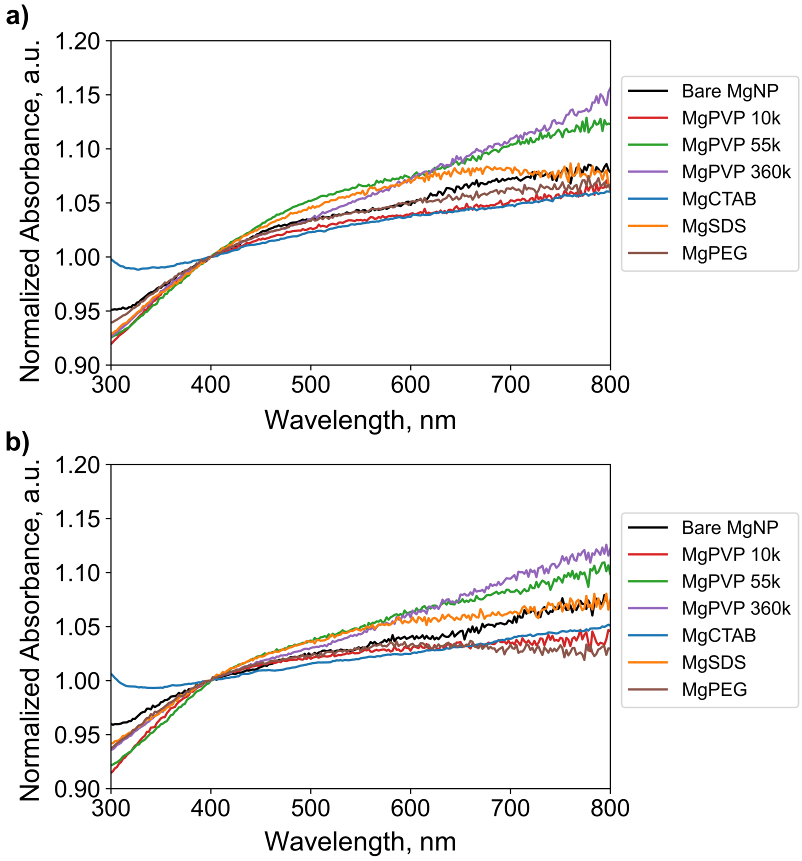

Figure S4. Extinction spectra of NP suspensions formed without and with the capping agents PVP (M_w = 10,000, 55,000, and 360,000), CTAB, SDS and PEG both (a) before and (b) after allowing NPs to sediment. Spectra are normalized at 400 nm and NPs were sonicated prior to acquisition in both cases.

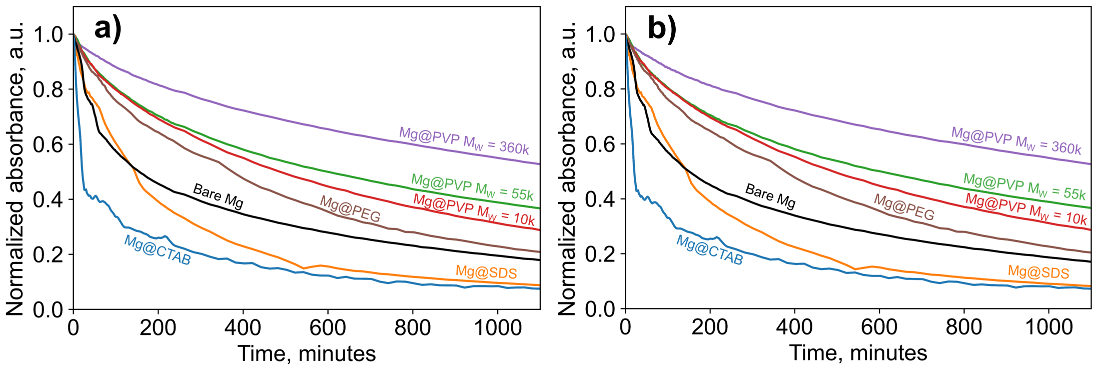

Figure S5. Single wavelength extinction traces over time showing colloidal stability of Mg NPs with varying capping agents at (a) 600 nm and (b) 750 nm.

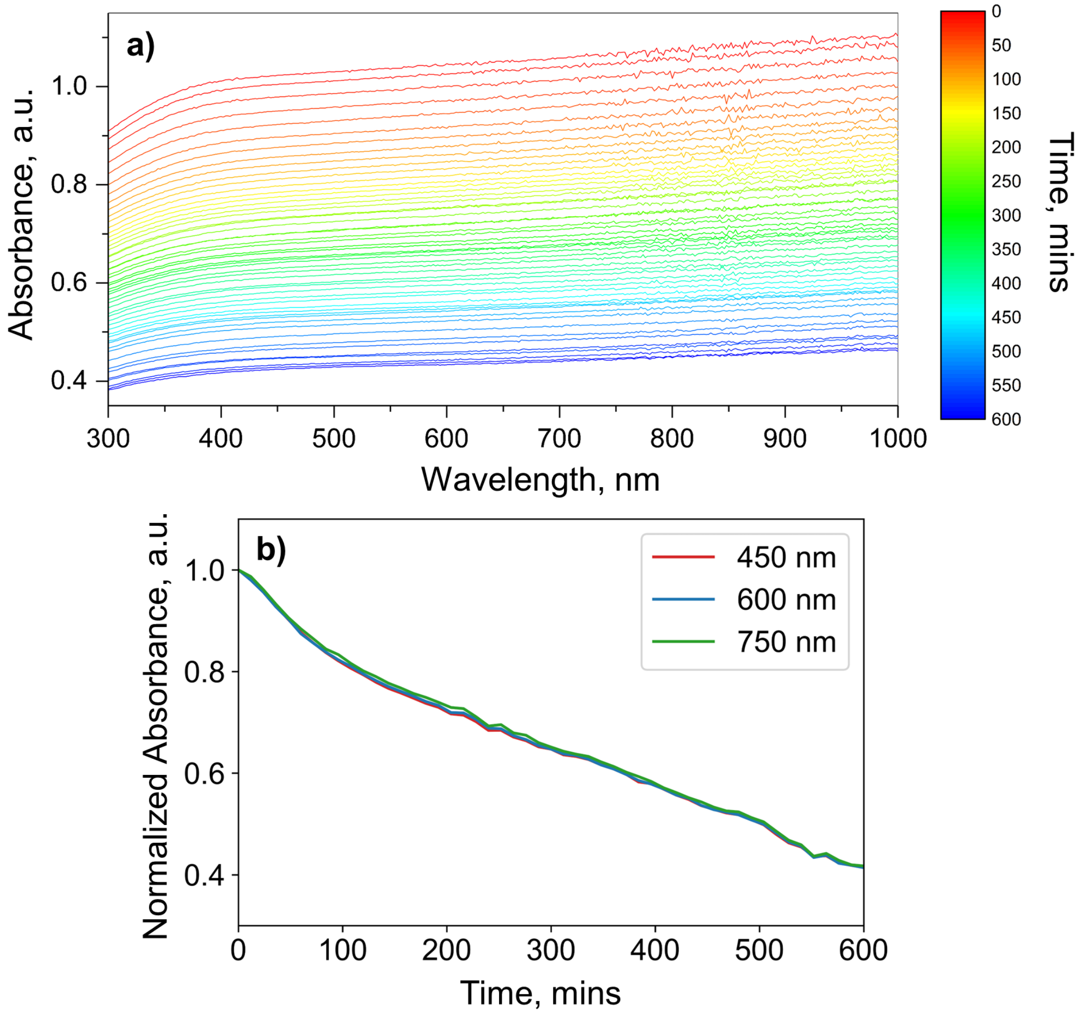

Figure S6. Time-resolved extinction spectra of Mg NPs synthesized in the presence of PVP (M_w = 10,000) showing (a) full 300-1000 nm wavelength scans acquired every 12 minutes for 10 hours and (b) single wavelength traces at 450, 600 and 750 nm taken from the 300-1000 nm scan. Particle suspension was sonicated prior to acquisition.

Table S4. Comparison of sedimentation tracked by absorption at 450, 600 and 750 nm between Mg NPs synthesized without and with the capping agents PVP, PEG, CTAB and SDS.

	450 nm		600 nm		750 nm	
Capping agent	Time to 50 % absorbance, mins	Relative absorbance after 19 hours, %	Time to 50 % absorbance, mins	Relative absorbance after 19 hours, %	Time to 50 % absorbance, mins	Relative absorbance after 19 hours, %
None	156	18	156	18	151	17
PVP, Mw = 10,000	483	28	502	28	502	28
PVP, Mw = 55,000	602	36	602	36	602	36
PVP, Mw = 360,000	>1140	53	>1140	52	>1140	52
PEG	383	20	383	20	383	20
CTAB	22	7	22	8	22	7
SDS	146	8	146	9	146	8

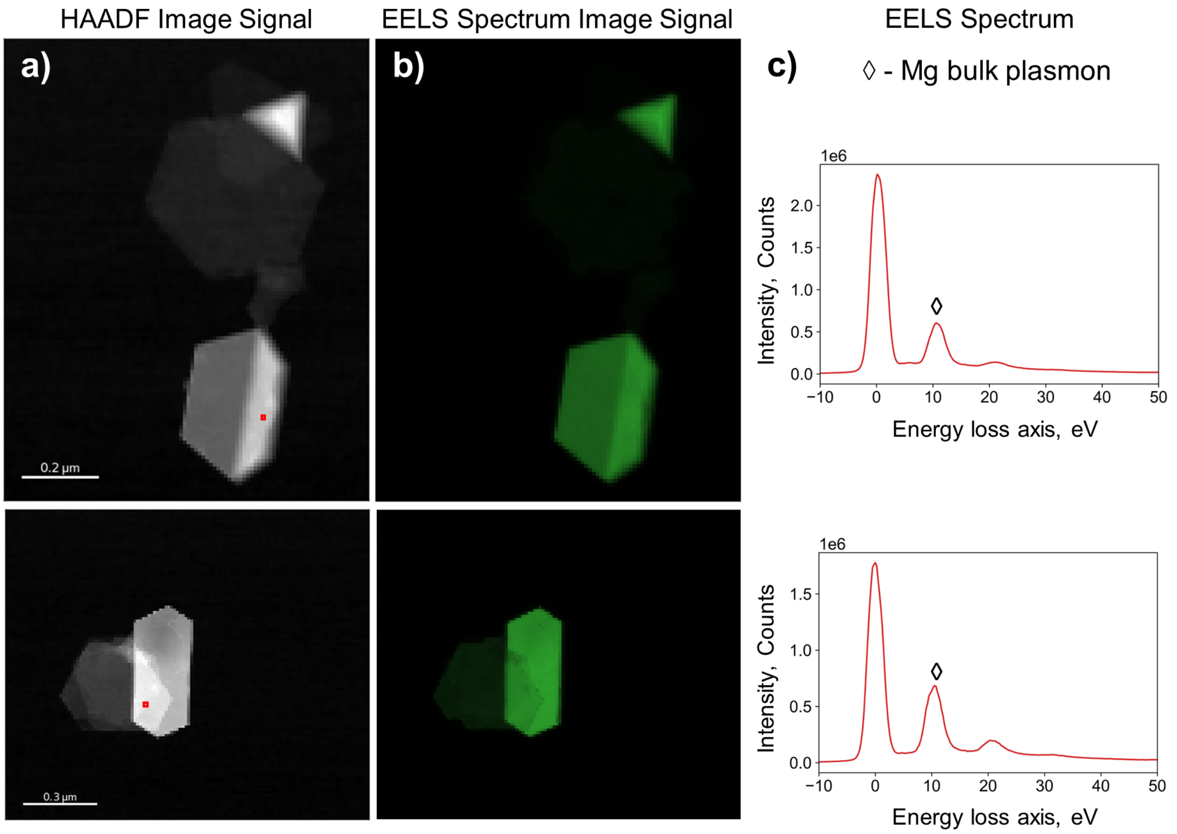

Figure S7. STEM-EELS maps of Mg particles synthesized in the presence of PVP M_w = 10,000. (a) HAADF images, corresponding (b) STEM-EELS Mg elemental maps from low-loss spectra, and (c) EELS spectra originating from the pixel highlighted by a red square in the HAADF images. Maps were generated using the 10.6 eV Mg bulk plasmon peak with the python-based Hyperspy software. The peak at ~21 eV results from double scattering of electrons by the Mg bulk plasmon.

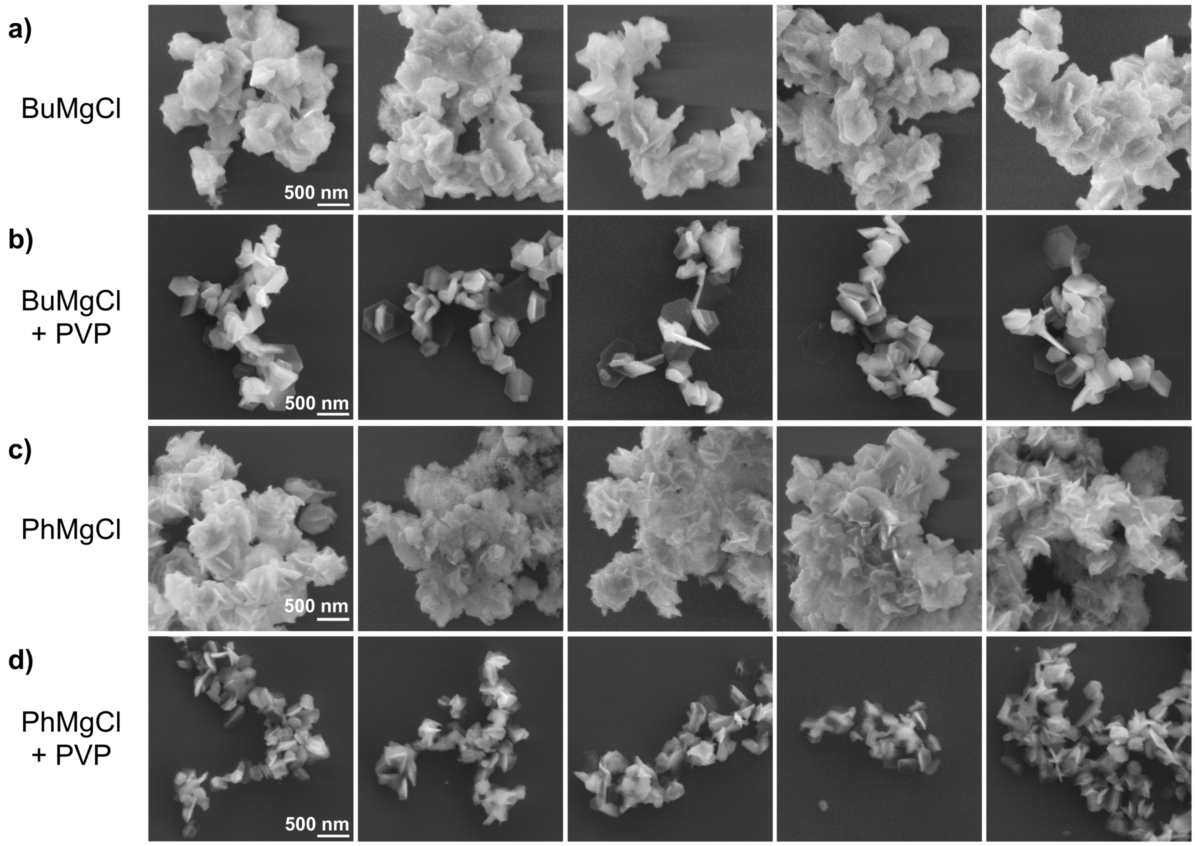

Figure S8. SEM images of Mg NPs synthesized from Grignard precursors: BuMgCl (a) without and (b) with PVP and PhMgCl (c) without and (d) with PVP M_w = 10,000.

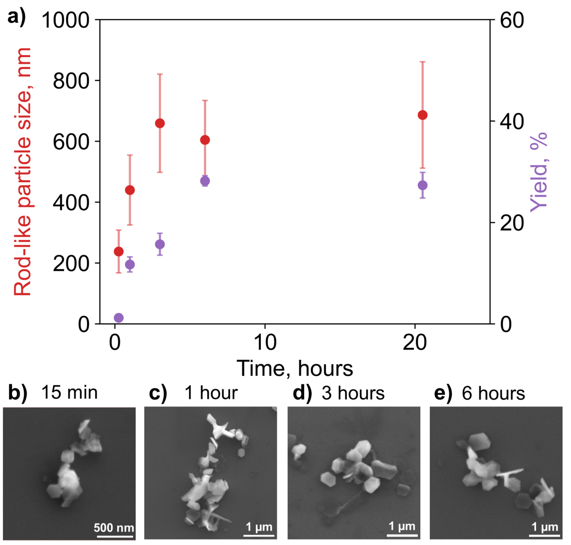

Figure S9. Effect of PVP on rod-like Mg NP growth from $MgBu_2$. (a) Rod-like NP size (red) and reaction yield (purple) as a function of time in the presence of PVP M_w = 10,000. The error bars report the standard deviation on the mean for 49 particles measured by SEM for 15 mins, 92 particles for 1 min and >150 particles in all other cases. SEM images of NPs synthesized in the presence of PVP for (b) 15 mins, (c) 1 hour, (d) 3 hours, and (e) 6 hours.

Table S5. Average particle size and standard deviations for Mg hexagonal platelets and rod-like particles and the number counted for syntheses using the precursors BuMgCl and PhMgCl both without and in the presence of PVP M_w = 10,000.

Precursor + capping agent	Average hexagonal platelet size, nm	Number of hexagonal platelets counted	Average rod-like particle size, nm	Number of rod-like particles counted
BuMgCl	400 ± 120	66	510 ± 50	3
BuMgCl + PVP	450 ± 150	50	400 ± 140	146
PhMgCl	280 ± 120	31	360 ± 110	19
PhMgCl + PVP	280 ± 80	54	320 ± 100	212

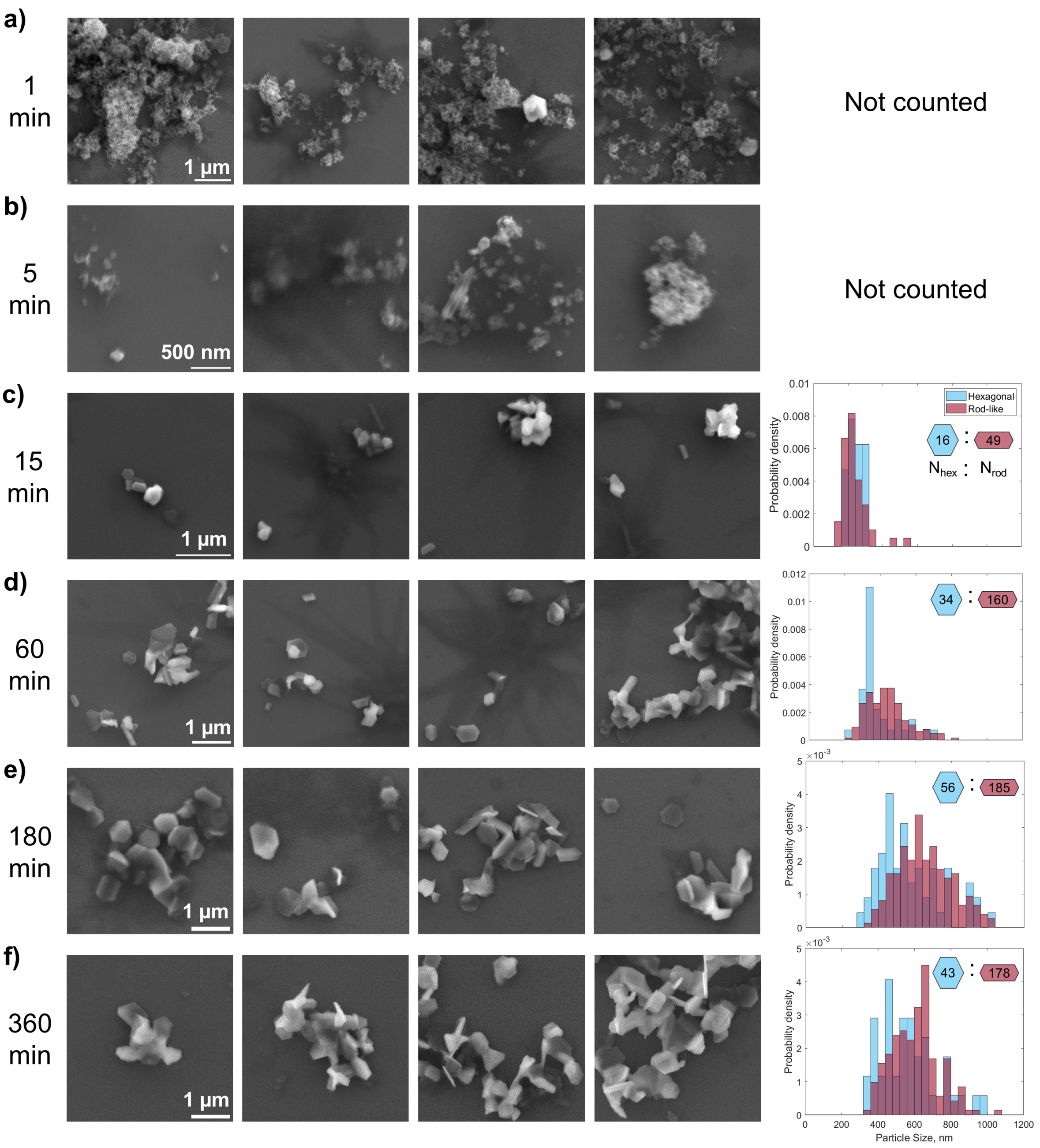

Figure S10. SEM images and, where applicable, NP size distribution histograms for hexagonal platelets and rod-like shapes for syntheses of Mg NPs in the presence of PVP M_w = 10,000 terminated at reaction times of (a) 1 minute, (b) 5 minutes, (c) 15 minutes, (d) 60 minutes, (e) 180 minutes and (f) 360 minutes. The NPs formed at the two shortest time could not reliably be measured due to the excess of organic contaminants present on their surface.

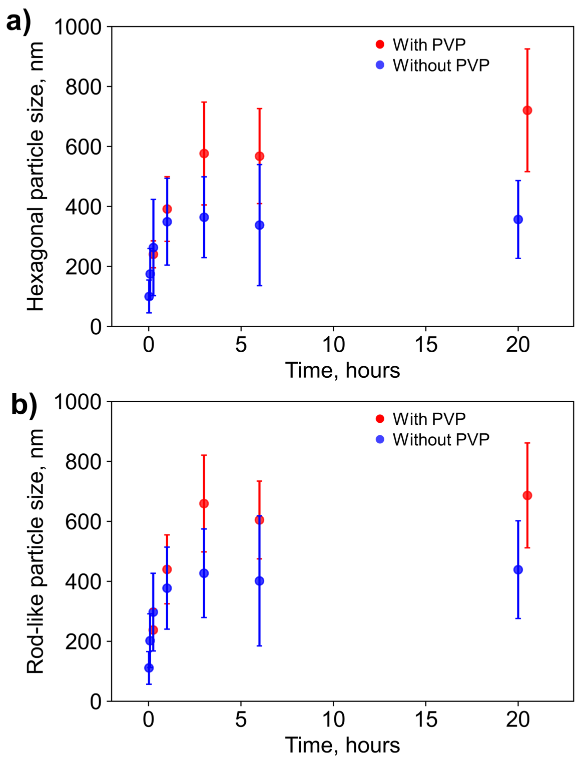

Figure S11. Effect of PVP on Mg NP growth. Comparison of average (a) hexagonal and (b) rod-like Mg NP s synthesized with (red) and without (blue) PVP (M_W = 10,000) present. Error bars show standard deviation of particle distributions. Data without PVP reproduced from [1] with permission.

Table S6. Average particle size and standard deviation for Mg NPs synthesized in the presence of PVP (M_w = 10,000) and the number counted for syntheses at varying reaction times.

Time, min	Average hexagonal platelet size, nm	Number of hexagonal platelets counted	Average rod-like particle size, nm	Number of rod-like particles counted
15	240 ± 45	16	240 ± 70	49
60	390 ± 110	34	440 ± 110	160
180	580 ± 170	56	660 ± 160	185
360	570 ± 160	43	600 ± 130	178
1230	720 ± 200	73	690 ± 170	236

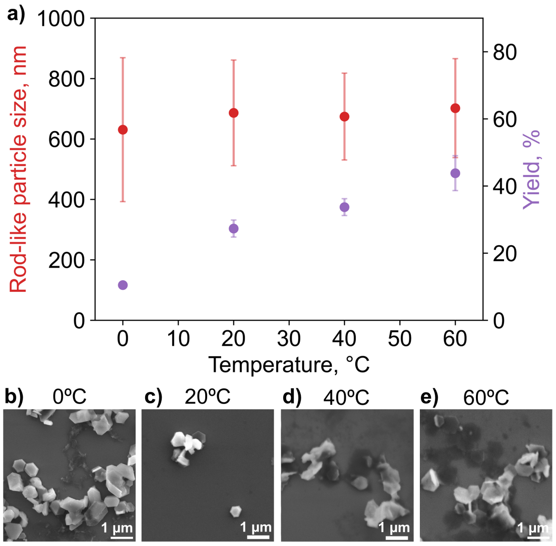

Figure S12. Effect of temperature on Mg NP formation from the reduction of $MgBu_2$ LiNapth in the presence of PVP $M_w = 10,000$. (a) Comparison of average rod-like NP size (red) and yield (purple) for NP synthesized in the presence of PVP $M_w = 10,000$ for ~20.5 hours at different temperatures and SEM images of NPs formed at (b) 0°C, (c) 20°C, (d) 40°C, and (e) 60°C. Error bars show standard deviation of particle distributions (red) or that of 3 yield measurements (purple).

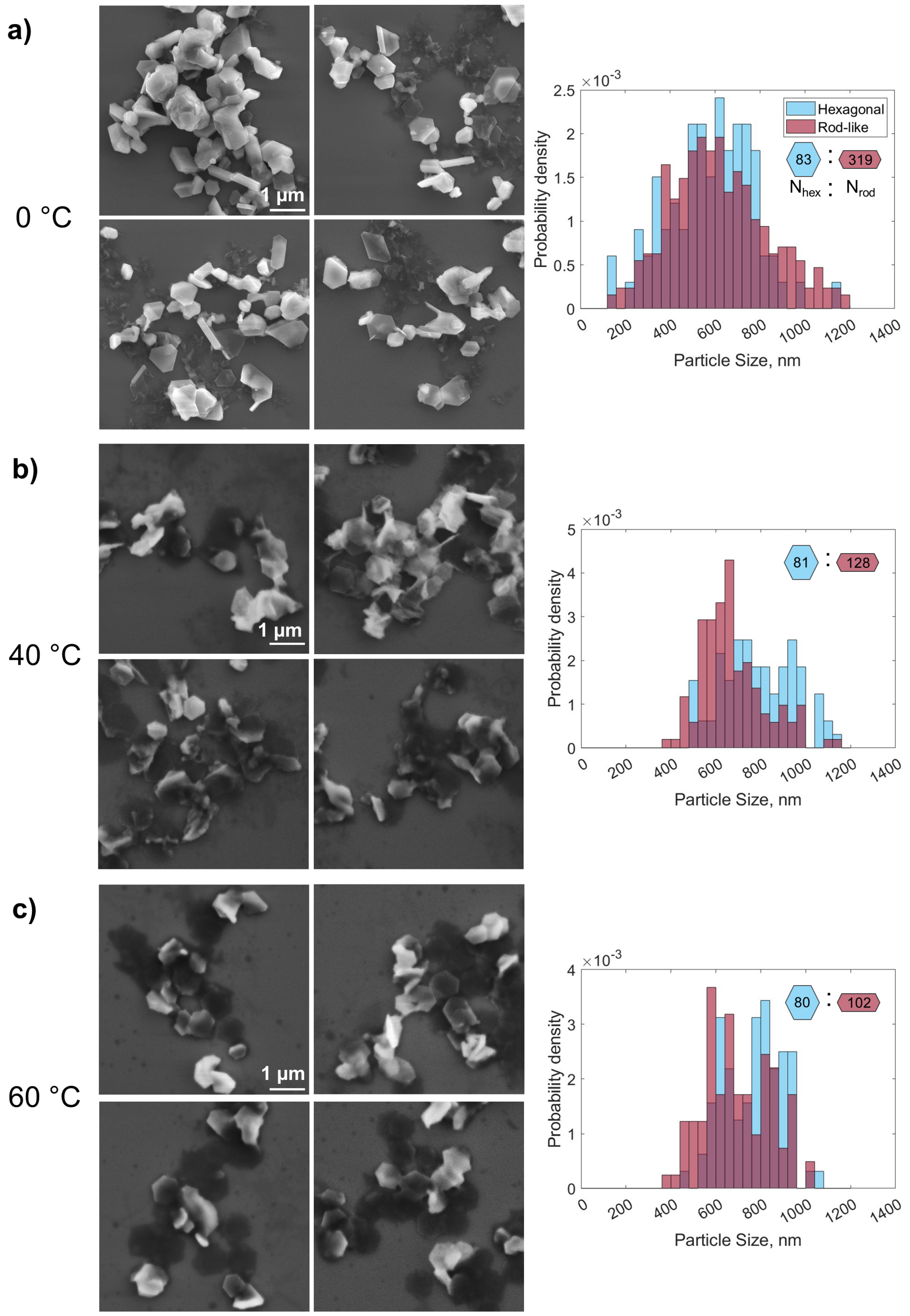

Figure S13. SEM images and NP size distribution histograms for hexagonal platelets and rod-like shapes for Mg NPs synthesized in the presence of PVP (M_W = 10,000) at temperatures of (a) 0 ºC, (b) 40 ºC and (c) 60 ºC. The same scale bar applies to all pictures for a given temperature.

Table S7. Average particle size and standard deviation for Mg NPs synthesized in the presence of PVP (M_w = 10,000) and the number counted for syntheses at varying reaction temperatures.

Temperature, °C	Average hexagonal platelet size, nm	Number of hexagonal platelets counted	Average rod-like particle size, nm	Number of rod-like particles counted
0	580 ± 190	83	630 ± 240	319
20	720 ± 200	68	690 ± 170	185
40	800 ± 170	81	670 ± 140	128
60	770 ± 130	80	700 ± 160	102

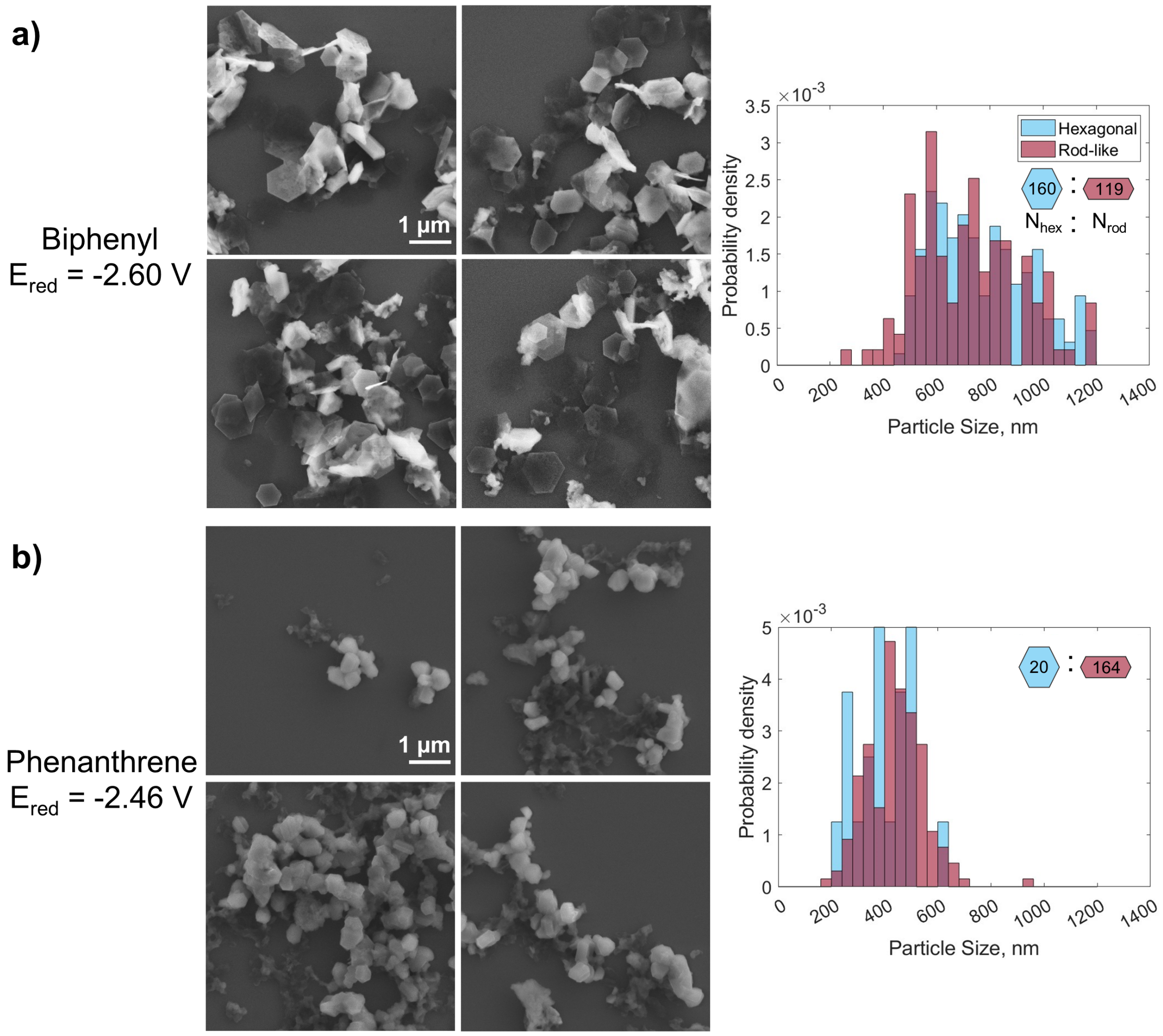

Figure S14. SEM images and NP size distribution histograms for hexagonal platelets and rod-like Mg NPs synthesized with the reducing agents (a) lithium biphenylide and (b) lithium phenanthride.

Table S8. Average particle size and standard deviation for Mg NPs synthesized in the presence of PVP (M_w = 10,000) and the number counted for syntheses using the electron carriers biphenyl, naphthalene and phenanthrene for generation of lithium arene reducing agents.

Electron carrier	Average hexagonal platelet size, nm	Number of hexagonal platelets counted	Average rod-like particle size, nm	Number of rod-like particles counted
Biph	800 ± 210	160	720 ± 200	119
Naph	720 ± 200	68	690 ± 170	185
Phen	400 ± 100	20	440 ± 110	164

References

1. Hopper, E. R.; Wayman, T. M. R.; Asselin, J.; Pinho, B.; Boukouvala, C.; Torrente-Murciano, L.; Ringe, E. Size Control in the Colloidal Synthesis of Plasmonic Magnesium Nanoparticles. *J. Phys. Chem. C* **2022**, *126* (1), 563–577. https://doi.org/10.1021/acs.jpcc.1c07544.
